# The role of gut microbiota-derived metabolites in modulating poultry immunometabolism

**DOI:** 10.3389/fphys.2025.1700406

**Published:** 2025-11-24

**Authors:** O. E. Oke, L. O. Fasasi, I. O. Opowoye, O. A. Akosile

**Affiliations:** 1 Department of Animal Physiology, Federal University of Agriculture, Abeokuta, Nigeria; 2 Department of Animal Production and Health, Federal University of Agriculture, Abeokuta, Nigeria

**Keywords:** immunometabolism, poultry, gut microbiota, metabolite, nutrition

## Abstract

The poultry sector is crucial to global food security, but it faces increasing challenges from heat stress, viral diseases, and restrictions on antibiotic use. These stressors highlight immunometabolism, the junction of immune function and metabolic pathways, as a crucial factor in determining the productivity and health of poultry. There is growing evidence that the gut microbiota is a dynamic metabolic organ that produces a diverse range of bioactive metabolites in addition to its function in nutritional digestion. The immunometabolism of poultry is significantly influenced by these microbiota-derived metabolites, including short-chain fatty acids, bile acid derivatives, amino acid catabolites, vitamins, and polyamines. Disease resistance, vaccination responsiveness, and stress adaptability are shaped by their modulation of intestinal barrier integrity, energy balance, oxidative stress resilience, and immune cell activation. This review summarises what is currently known about the functional diversity and composition of the gut microbiota in poultry, describes the concept of immunometabolism in birds, and assesses the mechanisms by which microbial metabolites regulate metabolic and immunological crosstalk. Prebiotics, probiotics, synbiotics, postbiotics, phytochemicals, and other nutritional and managerial interventions that improve advantageous metabolite profiles are given particular consideration. Applications to enhance poultry health, alleviate heat stress, reduce reliance on antibiotics, and promote sustainable production are also discussed. For mapping metabolite–immune interactions, emerging methods such as germ-free models, metabolomics, metagenomics, and systems biology approaches are emphasised as revolutionary. Metabolites produced by the gut microbiota are crucial to poultry immunometabolism and offer promising opportunities for precision nutrition and healthcare. Bridging the existing research gaps using integrative, multidisciplinary methods to promote sustainable and resilient poultry production is needed. This review centres on the mechanistic axis linking gut microbiota-derived metabolites to host immunometabolic regulation, tracing the pathway from metabolite generation through receptor activation and immune–metabolic reprogramming to measurable phenotypic outcomes in poultry.

## 1 Introduction

The poultry industry is one of the fastest-growing sectors in the global livestock industry, providing an affordable source of protein to meet the nutritional needs of a rapidly increasing global population (Kpomasse et al., 2021; Onagbesan et al., 2023; Oni et al., 2024). However, several stressors encumber sustainable poultry production globally. Some of the threats to health and productivity include disease outbreaks, climate change-related heat stress, and growing limitations on the use of in-feed antibiotics (Oke et al., 2021; Uyanga et al., 2023; Fushai et al., 2025). These challenges underscore the urgent need for innovative strategies that can enhance poultry’s innate resistance while maintaining productivity and food security.

Immunometabolism, which explores how metabolic pathways are altered to promote immune cell activation, proliferation, and defence mechanisms, represents an emerging paradigm in livestock production (Zhou et al., 2025). It is particularly important in poultry because of the constant need to balance efficient immune defence against pathogenic and environmental stressors with rapid development and effective feed conversion. Although immunometabolism has been thoroughly investigated in mammals, its mechanistic understanding in poultry remains limited. Consequently, where avian-specific data are scarce, insights from mammalian studies are referenced to illustrate conserved immunometabolic mechanisms, which are then discussed within the context of poultry physiology.

The gut microbiota plays an important role in poultry immunometabolism (Oni and Oke, 2025). The avian gut harbours a diverse microbial community that functions as metabolic “factories”, producing a wide array of bioactive compounds and aiding nutrient digestion. Microbial metabolites such as short-chain fatty acids, bile acid derivatives, amino acid catabolites, vitamins, and polyamines act as signalling molecules that link microbial activity with host immune and metabolic functions (Liu et al., 2022; Zhang Y. et al., 2023). These metabolites have a significant impact on poultry health, disease resistance, and overall productivity by modulating intestinal barrier integrity, energy utilisation, oxidative stress resilience, and immune cell differentiation (Oke et al., 2024).

Immunometabolism in poultry represents a dynamic interface between nutrient metabolism, immune regulation, and microbial activity within the gut ecosystem. While host-derived metabolites such as bile acids and amino acids play crucial roles in digestion and metabolic signalling, microbe-derived metabolites, including short-chain fatty acids (SCFAs), indoles, and secondary bile acids are increasingly recognised as essential regulators of immune and metabolic functions (Apajalahti and Vienola, 2016; Wickramasuriya et al., 2022; Fu et al., 2023). A clear distinction is therefore necessary between metabolites that originate from host digestive processes and those synthesised or modified by the gut microbiota, as both contribute differently to immunometabolic homeostasis.

Recent studies have emphasised the integrative nature of the host–microbiome–diet axis, which collectively determines immune competence and metabolic resilience in poultry. For example, dietary components such as non-starch polysaccharides, polyphenols, and amino acids can modulate microbial composition and activity, thereby influencing the profile of bioactive metabolites available to the host (Sugiharto, 2016; Makarewicz et al., 2021; Khasanah et al., 2024; Cheng et al., 2025). These metabolites, in turn, interact with host receptors such as G-protein coupled receptors (GPRs) and nuclear receptors (e.g., Farnesoid X Receptor (FXR)), mediating anti-inflammatory signalling, mucosal integrity, and energy partitioning. Understanding these interconnected metabolic and immunological pathways provides the foundation for exploring the roles of specific microbial and dietary metabolites in poultry health and productivity.

Despite their significance, the integration of metabolites produced by the gut microbiota into the framework of poultry immunometabolism remains limited (Zhao et al., 2025). Although direct evidence in poultry is still developing, a substantial part of the mechanistic understanding is extrapolated from mammalian research. Therefore, it is necessary to consolidate emerging knowledge, highlight molecular pathways, identify translational prospects, and consolidate current knowledge to enhance poultry resilience and performance.

The objective of this review is to provide a comprehensive overview of the role of gut microbiota-derived metabolites in modulating poultry immunometabolism. Specifically, the review outlines the functional diversity and composition of the gut microbiota of poultry. The review focuses on the mechanistic pathways through which gut microbiota-derived metabolites influence poultry immunometabolism. The discussion follows the axis of metabolite → receptor → immune/metabolic reprogramming → phenotypic outcomes, highlighting key molecular mediators, physiological relevance, and translational applications.

## 2 Gut microbiota in poultry: composition and functional diversity

A complex and dynamic microbial environment in the gastrointestinal tract of poultry is essential for immune system development, metabolic regulation, and nutrient digestion (Yadav and Jha, 2019; Aruwa et al., 2021). Although host species, production method (broilers vs. layers), food, and environmental factors affect the microbiota’s composition, a few phyla continuously predominate. The most prevalent bacteria in broilers and layers are Firmicutes (e.g., *Clostridium* and *Lactobacillus*) and Bacteroidetes, which aid in carbohydrate fermentation and the synthesis of short-chain fatty acids (SCFAs) (Pan and Yu, 2013; Liu et al., 2021).

Although they are less common, actinobacteria, particularly Bifidobacterium, play crucial roles in immunological regulation and polysaccharide metabolism. Although they are usually present in low concentrations, proteobacteria can increase in response to stress or dysbiosis and are commonly associated with pathogenic species such as *Salmonella* and *Escherichia coli*. According to comparative research, layers maintain a relatively stable microbial population focused on lifespan and egg production, whereas broilers, selected for fast growth, typically have higher proportions of Firmicutes associated with energy harvest (Khan et al., 2020; Ricke et al., 2022; Bajagai et al., 2024).

Immediately after hatching, the gut microbiome starts to develop. As birds age, *Clostridium*, *Bacteroides* and *Lactobacillus* progressively replace or balance the early colonisers, such as *Enterococcus* and *Escherichia*, which establish within the first few days (Pan and Yu, 2013; Al Hakeem et al., 2023). The microbial community stabilises by 3–4 weeks of age, but it remains susceptible to environmental changes. When broilers reach market age, their gut microbiota usually develops a high fermentative capacity, which is ideal for extracting energy from feed non-starch polysaccharides and complex carbohydrates (Al Hakeem et al., 2023).

The functional diversity and composition of the intestinal microbiota of poultry are influenced by various variables. The most important factor is diet, as microbial fermentation patterns are shaped by components such as grains, fibre, and protein sources (Pan and Yu, 2013). Since various breeds have unique microbiological fingerprints, genetics also plays a role. The microbial equilibrium can be altered by environmental factors (temperature, stocking density, litter type, and housing) and stressors (transport, immunisation, and heat stress), which often favour opportunistic pathogens. Historically, the use of antibiotics has altered gut microbial ecosystems, decreased their diversity, and increased their susceptibility to resistance (Lathakumari et al., 2024). On the other hand, probiotic, prebiotic, and synbiotic supplementation increases microbial metabolic outputs and encourages the growth of beneficial bacteria.

The gut microbiota of poultry functions as a bioreactor, producing a variety of metabolites from indigestible food sources. SCFAs, including butyrate, propionate and acetate, are produced during the fermentation of complex carbohydrates and are used by enterocytes as energy sources and immune response modulators (Liu et al., 2021). Compounds, such as nitric oxide, polyamines and indole precursors, are produced by microbial amino acid metabolism and have a wide range of impacts on intestinal health and systemic immunity (Bui et al., 2023). The microbiota also plays a role in vitamin production and bile acid metabolism, highlighting its role as a metabolic organ closely related to the health and performance of poultry. While knowledge of microbial community structure provides valuable context, the immunometabolic effects of poultry microbiota are ultimately determined by the functional metabolites they produce.

### 2.1 Interactions between microbiota-derived metabolites and age-dependent dynamics

Gut microbiota-derived metabolites do not act in isolation; rather, they engage in synergistic and antagonistic interactions that collectively influence poultry immunometabolism. Short-chain fatty acids (SCFAs), bile acids, and tryptophan metabolites can modulate immune responses, gut barrier integrity, and energy metabolism in complementary or opposing ways. For instance, SCFAs may enhance bile acid signalling, while certain tryptophan derivatives can either potentiate or inhibit SCFA-mediated immune modulation, depending on host physiological conditions (Zhang W. et al., 2023; Yan et al., 2023; Tang et al., 2025). Importantly, the production and utilisation of key metabolites are influenced by both bird age and gut region. Early post-hatch stages correspond with rapid immune and microbial maturation, particularly in the caeca, which serve as the primary site of short-chain fatty acid (SCFA) production. Segment-specific receptor expression, such as Free Fatty Acid Receptor (FFAR)2/3 in the caeca and ileum, and FXR/Takeda G-Protein-Coupled Bile Acid Receptor 5 (TGR5) in the small intestine, further underscores the need for age- and site-targeted nutritional interventions.

The immunometabolic landscape is highly dynamic and age-dependent. Hatchlings typically exhibit lower microbial diversity and metabolite concentrations, whereas growers and finishers show progressive increases in both diversity and metabolite abundance (Montso et al., 2022; Akram et al., 2024; Wu et al., 2025). These age-dependent variations have important implications for stage-specific nutritional and microbiota-targeted interventions, enabling tailored strategies to optimise gut health, immune function, and overall growth performance at each developmental stage. During early post-hatch development, rapid shifts in gut microbial colonisation correspond with changes in metabolite production and immune maturation. The transition from yolk-dependent nutrition to exogenous feeding alters the availability of microbial substrates and the expression of host metabolic genes, influencing both innate and adaptive immune responses. Studies such as Proszkowiec-Weglarz et al. (2020), Al Hakeem et al. (2023) and Gupta et al. (2025) have demonstrated that these developmental transitions shape the crosstalk between metabolic and immune pathways, highlighting that immunometabolic regulation in poultry must be viewed as a time-dependent, co-adaptive process involving diet, host physiology, and microbial metabolism.

## 3 Concept of immunometabolism in poultry

The dynamic interaction between immune activity and cellular metabolism is known as immunometabolism, and it emphasises how metabolic pathways are composed to satisfy the biosynthetic and energetic requirements of immunological activation. Depending on their functional condition, immune cells in poultry, like those in other animals, change their metabolic profile. T lymphocytes, macrophages and heterophils are immune cells that rapidly undergo metabolic reprogramming in response to infections or stress signals to support proliferation, cytokine production, and antimicrobial defence (Darweesh et al., 2025).

The main mechanism facilitating prompt immune activation is glycolysis. To enable quick antimicrobial responses, activated macrophages and heterophils depend on glycolysis to provide quick bursts of ATP and biosynthetic intermediates (Darweesh et al., 2025). In poultry, as in mammals, metabolic reprogramming of immune cells represents a hallmark of immune activation. Activated macrophages rely predominantly on glycolysis for rapid ATP generation, whereas long-term immune functions depend on oxidative phosphorylation (OXPHOS). The dynamic shift between glycolysis and OXPHOS therefore determines whether responses are inflammatory, regulatory, or memory-oriented. This metabolic change, frequently referred to as the “Warburg effect,” prioritises speed over efficiency, ensuring that immune effector functions are initiated as soon as possible during acute assaults. On the other hand, long-term immunological responses depend on oxidative phosphorylation. The long-term energy needed for memory cell growth, immune function regulation, and inflammation resolution is provided by mitochondrial metabolism (Tandon et al., 2021). Thus, whether immune responses are memory-oriented, regulatory, or pro-inflammatory depends on the ratio of glycolysis to oxidative phosphorylation.

Classic works by Klasing (1998), Klasing (2007) provided insights into the energetic costs of immune activation in poultry and the metabolic trade-offs between growth and defence. These studies established the conceptual framework for understanding immunometabolic resource allocation in birds, highlighting the influence of nutrient availability and infection on metabolic flux. Immunometabolic control is further integrated with amino acid metabolism. Nitric oxide, a vital antibacterial chemical produced by macrophages, uses arginine as a substrate. Tryptophan degradation via the kynurenine route affects mucosal homeostasis and immunological tolerance, while glutamine serves as a fuel source for lymphocyte proliferation and facilitates nucleotide synthesis (Badawy, 2017). The strength and persistence of immune responses are directly influenced by the availability and utilisation of these amino acids.

The close relationship between feed efficiency, growth rate, and metabolic condition makes immunometabolism in poultry even more important. Immune activation creates a crucial trade-off between disease resistance and production by diverting resources away from growth (Dadfar et al., 2023). This trade-off is worsened by stressors such as exposure to pathogens, vaccinations, or extreme temperatures, which frequently hinder growth and induce immunological responses that are metabolically taxing (Kpomasse et al., 2021; Kpomasse et al., 2023; Oke et al., 2024b). For poultry to be as resilient as possible, immunometabolism must be understood and carefully modulated.

The impact of microbial and nutritional pathways on immunometabolic processes is a new field of study. Gut microbiota-derived metabolites, such as amino acid catabolites, bile acid derivatives and short-chain fatty acids, have been demonstrated to alter immune cell metabolism in ways that balance development and defence (Liu et al., 2022). These interactions support the larger objective of sustainable poultry production by providing plausible pathways to improve birds’ welfare and productivity without relying heavily on antibiotics.

**FIGURE 1:**
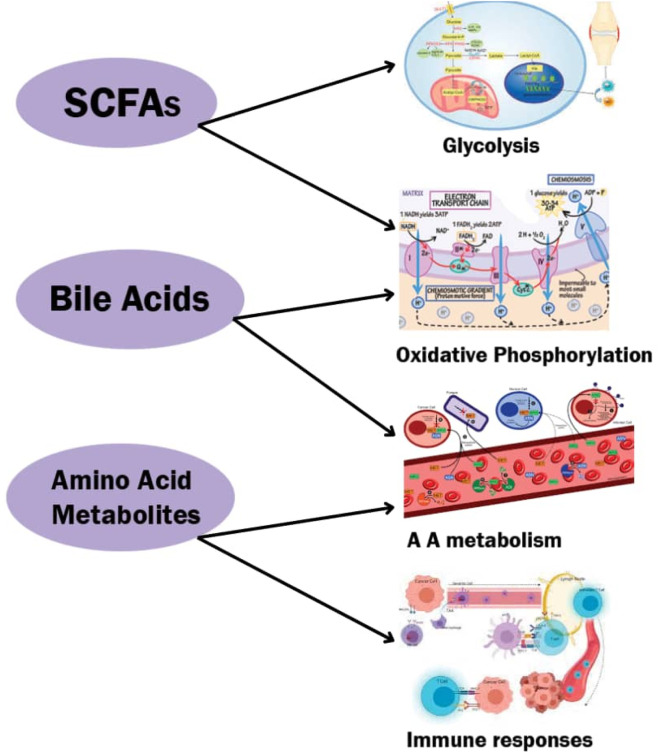
Key metabolic pathways influenced by microbial metabolites. Schematic illustration showing how different classes of gut microbiota-derived metabolites modulate cellular energy metabolism and immune responses in poultry. SCFAs (short-chain fatty acids): Regulate glycolytic pathways by providing alternative energy substrates and enhancing ATP generation in immune and epithelial cells. Bile acids: Influence oxidative phosphorylation by modulating mitochondrial function and reactive oxygen species balance through activation of FXR and TGR5 receptors. Amino acid metabolites: Participate in amino acid metabolism (A.A. metabolism), supporting protein synthesis, nitrogen recycling, and immune cell proliferation. Immune responses: The combined effects of these metabolites regulate cytokine production, T-cell differentiation, and macrophage activation, maintaining immune–metabolic homeostasis. Arrows: Indicate the direction of metabolic regulation between metabolites and corresponding cellular processes.

The major classes of microbial metabolites—short-chain fatty acids (SCFAs), bile acid derivatives, and amino acid catabolites—that interact with host metabolic and immune pathways are illustrated in Figure 1. SCFAs enhance gut barrier integrity, serve as energy substrates for enterocytes, and induce *regulatory T cells* differentiation. Bile acid metabolites act through FXR and TGR5 receptors to regulate lipid and glucose metabolism while modulating inflammatory signalling. Amino acid-derived metabolites, such as arginine (→ nitric oxide), tryptophan (→ indole derivatives and kynurenine), and glutamine, support immune effector functions, tolerance, and lymphocyte proliferation. These metabolites orchestrate poultry immunometabolism by integrating nutrient utilisation, immune modulation, and stress resilience, ultimately improving health, productivity, and welfare.

## 4 Microbiota-derived metabolites and their immunometabolic roles

Several metabolites produced by gut microbial communities act as molecular links between host physiology, microbiota, and nutrition. These substances are key modulators of immunometabolism in poultry, influencing oxidative balance, immune responses, and nutrient utilisation. Major microbiota-derived metabolites, their microbial sources, and roles in poultry immunometabolism are shown in Table 1. Key groups of metabolites produced by the microbiota and their physiological significance are highlighted below.

**TABLE 1:** Major microbiota-derived metabolites, their microbial sources, and roles in poultry immunometabolism.

Metabolite class	Key examples	Microbial source/Pathway	Immunometabolic roles in poultry	References
Short-chain fatty acids (SCFAs)	Acetate, propionate, butyrate	Fermentation of non-digestible carbohydrates by *Clostridium*, *Ruminococcus*, and *Bacteroides*	Enhance epithelial barrier, fuel enterocytes, modulate tregs, anti-inflammatory effects, and oxidative stress resilience	Duan et al. (2023), Pant et al. (2023), Chen et al. (2025)
Bile acid metabolites	Deoxycholic acid, lithocholic acid	Microbial deconjugation and dehydroxylation of primary bile acids	Regulate lipid metabolism, energy balance, FXR/TGR5-mediated immune signalling	Huang, et al. (2023), Larabi et al. (2023)
Amino acid-derived metabolites	Nitric oxide, indole, kynurenine, polyamines	Arginine → NO; tryptophan → indole derivatives and kynurenine; ornithine/Glutamine → polyamines	Immune tolerance, macrophage polarization, Th1/Th2 balance, lymphocyte proliferation	Gantner et al. (2020)
Vitamins and cofactors	Folate, vitamin B12, biotin, riboflavin	Synthesised by *Lactobacillus*, *Bifidobacterium*, *Clostridium*	Support antioxidant defence, nucleotide synthesis, one-carbon metabolism, DNA repair	Tumilaar et al. (2023), Yu et al. (2025)
Other metabolites	Conjugated linoleic acids, microbial peptides	Produced by diverse gut bacteria	Pathogen inhibition, immune tolerance, metabolic efficiency	Piqueras et al. (2021)

### 4.1 Short-chain fatty acids (SCFAs)

In the ceca, microbial fermentation of resistant starches and dietary fibres produces SCFAs, mainly acetate, propionate, and butyrate. The physiology of poultry is impacted by these compounds in a variety of ways.

- Integrity of the epithelium: Butyrate increases the expression of tight junction proteins, which lowers intestinal permeability and the movement of pathogens (Tabat et al., 2020).
- Immunomodulation: SCFAs encourage the development of *regulatory T cells* (Treg), which support mucosal tolerance and anti-inflammatory reactions (Park et al., 2014; Duan et al., 2023).
- Energy supply: Acetate and propionate support systemic energy metabolism, while butyrate is the primary fuel source for enterocytes (Lange et al., 2023).
- Resilience to oxidative stress: SCFAs enhance antioxidant capacity, lower the expression of inflammatory cytokines, and reduce tissue damage in heat-stressed broilers (Al-Khalaifah et al., 2025).

### 4.2 Bile acid metabolites

Microbes in the poultry’s gut convert the liver’s primary bile acids into secondary bile acids, which then interact with host receptors such as Takeda G-protein receptor 5 (TGR5) and farnesoid X receptor (FXR) (Pathak et al., 2017). These signalling pathways control the following: immune signalling, which includes the regulation of macrophage polarisation and inflammatory cytokines; glucose homeostasis, which may improve metabolic efficiency; and lipid and energy metabolism, which support effective feed conversion and growth.

Bile acid metabolism is especially crucial for maintaining nutrient absorption and influencing immunological responses, since commercial poultry diets contain a significant amount of dietary fat.

### 4.3 Amino acid-derived metabolites

Metabolites with significant immunometabolic roles are produced by the host and microbial enzymes that break down amino acids.

- Nitric oxide (NO), a crucial immune effector molecule involved in pathogen destruction, vasodilation, and the control of inflammatory tone, is produced from arginine (Gantner et al., 2020)
- Tryptophan: Catabolised into kynurenine, which enhances immunological tolerance and reduces excessive inflammation, or indole derivatives, which strengthen the function of the epithelial barrier.
- Glutamate: Promotes nucleotide production and offers a vital energy source for lymphocytes that divide quickly.

These pathways demonstrate the dual function of amino acids as immunomodulatory substrates and nutrition.

### 4.4 Vitamins and cofactors

Riboflavin, folate, biotin and B12 are among the B vitamins that are biosynthesised by the intestinal microbiota of poultry. These substances are necessary for.

- Antioxidant defence mechanisms, which shield immune cells from oxidative damage (Tumilaar et al., 2023; Oke et al., 2025).
- The production of DNA and RNA, which promotes the quick growth of lymphocytes during immunological reactions (Treichel et al., 2025).
- One-carbon metabolism, which affects how immune cells are epigenetically regulated (Ducker and Rabinowitz, 2017).

Microbial contributions may offer localised benefits at the gut–immune interface, even though poultry diets are usually supplemented with vitamins.

### 4.5 Other emerging metabolites

In addition to well-known substances, the field of poultry science is increasingly focusing on several newly discovered groups of microbial metabolites.

Polyamines, such as putrescine, spermidine, and spermine, control immunological tolerance, mucosal healing, and epithelial proliferation (Nakamura and Matsumoto, 2025).

Microbial peptides function as natural antimicrobials, inhibiting pathogenic colonisation and regulating innate immunity. Microbial biohydrogenation produces conjugated linoleic acids (CLAs), which have anti-inflammatory and lipid-metabolising properties (Salsinha et al., 2018).

These newly discovered metabolites demonstrate the unexplored diversity of microbial compounds that may have functions in immunometabolism and general health in birds.

## 5 Mechanistic insights: how metabolites influence poultry immunometabolism

**FIGURE 2:**
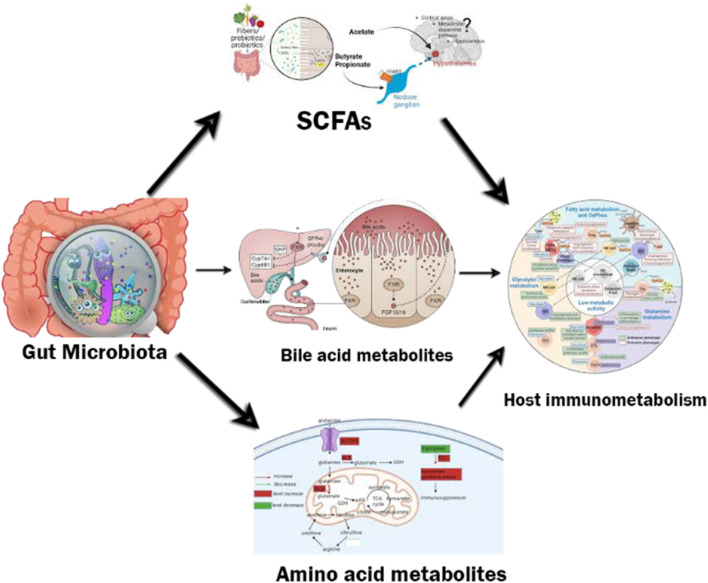
Microbiota–metabolite–immune crosstalk in poultry. Schematic illustration showing the dynamic interactions between gut microbiota, their metabolites, and the host immunometabolic system. Gut microbes ferment dietary components, such as fibres, prebiotics, and probiotics, to produce short-chain fatty acids (SCFAs), which modulate host energy metabolism, gut barrier function, and immune signalling. Microbial conversion of primary to secondary bile acids regulates farnesoid X receptor (FXR) and fibroblast growth factor (FGF15/19) pathways, influencing lipid metabolism and mucosal homeostasis. Amino acid metabolites (e.g., glutamine, arginine) support immune cell activation and oxidative balance by fueling the tricarboxylic acid (TCA) cycle. These metabolites form an integrated communication network that coordinates metabolic and immune responses to maintain health and performance in poultry. Gut Microbiota: Primary source of fermentation and metabolite synthesis. SCFAs (acetate, propionate, butyrate): Microbial metabolites that provide energy and modulate immune pathways. Bile acid metabolites: Secondary bile acids produced by microbial enzymes; regulate FXR/TGR5 signalling. Amino acid metabolites: Glutamine and arginine derivatives that influence immune metabolism and cytokine synthesis. Host immunometabolism: Integration of microbial and host metabolic signalling networks governing immunity and nutrient utilisation. Arrows: Indicate direction and nature of interaction among components.

The effects of microbiota-derived metabolites on poultry health and productivity are mediated through various cellular and molecular mechanisms. These mechanisms integrate microbial signals with host immune, metabolic, and endocrine networks, providing a systems-level understanding of immunometabolic regulation. Figure 2 shows microbiota–metabolite–immune crosstalk in poultry. This schematic figure illustrates the interconnected roles of diet, gut microbiota, microbial metabolites, and host responses in poultry. Dietary inputs, such as fibre, protein, and phytochemicals, are metabolised by the gut microbiota (Firmicutes, Bacteroidetes, Actinobacteria, Proteobacteria) to generate bioactive metabolites, including short-chain fatty acids (SCFAs), bile acid derivatives, amino acid catabolites, vitamins, and polyamines. These metabolites function as signalling molecules that influence both the host immune system (e.g., T cells, macrophages, NK cells) and metabolic processes (e.g., lipid, glucose, and amino acid metabolism). The outcome of this cross-talk is modulation of immunometabolism, enhancing gut barrier integrity, regulating inflammatory responses, and improving stress resilience.

Collectively, these signalling events illustrate the integrated ‘metabolite–receptor–immune–metabolic’ axis through which gut microbiota influence poultry physiology. This mechanistic framework underpins observed improvements in feed efficiency, vaccine responsiveness, and heat stress tolerance in birds receiving microbiota-modulating diets.

### 5.1 Epigenetic regulation

Numerous metabolites directly affect gene expression in immunological and metabolic pathways by acting as epigenetic modifiers.

- Histone acetylation: By blocking histone deacetylases (HDACs), butyrate and other SCFAs enhance the transcription and acetylation of genes related to barrier function and anti-inflammatory responses (Du et al., 2024).
- DNA methylation: Cytokine production and immune cell differentiation are modulated by the microbial synthesis of folate and other one-carbon metabolites, which affect DNA methylation patterns (Claycombe et al., 2015).

Metabolites produced by the microbiome can alter immune response and stress tolerance over time through various mechanisms.

### 5.2 Cellular signalling pathways

Short-chain fatty acids activate G-protein-coupled receptors FFAR2 (GPR43), FFAR3 (GPR41), and the Hydroxycarboxylic Acid Receptor (HCAR)2 (GPR109A) on intestinal and immune cells, regulating cytokine expression and energy use. Similarly, bile acid metabolites engage nuclear receptors FXR and TGR5, influencing glucose and lipid metabolism while suppressing inflammation. Tryptophan-derived indoles activate the aryl hydrocarbon receptor, promoting mucosal homeostasis. Microbial metabolites activate pathways that connect immunity and metabolism by interacting with nuclear and cell surface receptors on the host.

- G-protein coupled receptors (GPR41, GPR109A and GPR43): These receptors control energy consumption, leukocyte trafficking, and cytokine production when they are activated by SCFAs (Parada Venegas et al., 2019).
- Bile acid receptors (FXR, TGR5): Secondary bile acids attach to these receptors and affect glucose balance, inflammatory signalling, and lipid metabolism (Chiang and Ferrell, 2020).
- Toll-like receptors (TLRs): Many compounds from microorganisms bind to TLRs, triggering innate immune responses (Lester and Li, 2013).

Microbial products act as molecular messengers between host physiology, microorganisms, and diet through receptor-mediated pathways.

Studies (Kogut, 2019; Kogut and Arsenault, 2016; Kogut, 2022) advanced the concept of immunometabolic crosstalk between the chicken gut microbiota and host immune pathways. Their work demonstrated how microbial-derived short-chain fatty acids and other metabolites modulate inflammatory tone, macrophage metabolism, and epithelial integrity, providing mechanistic evidence for host–microbiota–nutrient integration in poultry.

### 5.3 Immune cell modulation

Metabolites from the microbiota directly control immune cell activation, differentiation, and polarisation.

- T cells: SCFAs lower the risk of severe inflammation by balancing Th1/Th2 responses and encouraging the development of *regulatory T cells* (Duan et al., 2023).
- Macrophages: By influencing macrophage polarisation towards anti-inflammatory M2 phenotypes, butyrate and bile acid derivatives improve tolerance and tissue healing (Duan et al., 2023).
- Natural killer (NK) cells: Several metabolites formed from amino acids increase the cytotoxicity of NK cells, strengthening defence against intracellular and viral infections (Naidoo and Altfeld, 2024). These demonstrate how metabolites adjust immunity to strike a balance between growth efficiency and pathogen removal.

### 5.4 Cross-talk with the endocrine system

Another layer of immunometabolic regulation, influenced by microbial metabolites, is provided by the endocrine system.

- Stress hormones: By preserving barrier integrity and regulating stress reactions, SCFAs and indole derivatives can reverse the effects of heat stress and corticosterone production, which change gut permeability and immunological suppression (Yan et al., 2023; Nie et al., 2024).
- Metabolic hormones: Insulin sensitivity and energy expenditure are influenced by bile acid signalling via FXR and TGR5, which also indirectly impacts immunological function (Chiang, 2017).
- Neuroimmune interactions: Microbial metabolism is linked to stress resilience and behaviour through the influence of tryptophan metabolites (such as serotonin precursors) on gut–brain axis signalling (Xu et al., 2025).

## 6 Management and nutritional interventions to harness microbiota-derived metabolites

Effective modulation of poultry immunometabolism through gut microbiota-derived metabolites requires evidence-based management and nutritional strategies. Among these, prebiotics, probiotics, and precision nutrition approaches have shown substantial promise in optimising gut microbial activity, metabolite production, and host resilience under environmental or physiological stressors. Opportunities to strategically control microbial activity for better poultry health and production arise from the gut microbiota’s capacity to create compounds with immunometabolic activities. Several dietary and management strategies can mitigate stress responses, increase the production of healthy metabolites, and reduce the need for antibiotics. The efficacy of microbiota-targeted interventions depends on dosage, duration, and environmental context. Table 2 summarises the management and nutritional interventions to harness microbiota-derived metabolites in poultry.

**TABLE 2:** Nutritional and management interventions to harness microbiota-derived metabolites in poultry.

Intervention	Mode of action	Impact on metabolites	Potential benefits for immunometabolism	References
Prebiotics (e.g., inulin, MOS, FOS)	Provide fermentable substrates for beneficial microbes	Increases SCFAs (butyrate, acetate)	Improved gut barrier, treg activation, and anti-inflammatory effects	Baurhoo et al. (2007)
Probiotics (*Lactobacillus*, *Bacillus*)	Modulate microbial composition and activity	Enhanced vitamin B production, altered SCFA profiles	Balanced immune response, pathogen suppression	Mountzouris, et al. (2007)
Synbiotics	Synergistic action of prebiotics + probiotics	Optimised SCFA production and metabolite diversity	Stronger immune stimulation, metabolic efficiency	Awad, et al. (2009)
Postbiotics (e.g., direct SCFA supplementation)	Delivery of microbial metabolites without live microbes	Direct increase in key metabolites	Improved oxidative stress resilience, immune cell activation	Aguilar-Toalá, et al. (2018)
Phytochemicals (polyphenols, essential oils)	Modulate microbial metabolism and inhibit pathogens	Promote indole derivatives, SCFAs	Antioxidant effects, immune tolerance	Greathead (2003)
Fermented feeds	Supply preformed metabolites, reshape gut microbiota	SCFAs, polyamines, vitamins	Enhanced gut health, metabolic efficiency	Missotten, et al. (2015)
Precision nutrition	Tailored diets for genotype/environment	Targeted metabolite shifts (e.g., tryptophan, glutamine)	Optimised growth–immunity balance	Apajalahti, and Vienola. (2016)

### 6.1 Prebiotics

Prebiotics such as fructooligosaccharides (FOS), inulin, and mannan oligosaccharides (MOS) stimulate the proliferation of beneficial bacteria, particularly *Lactobacillus* and Bifidobacterium spp., thereby enhancing SCFA production and improving gut barrier function. Beneficial microbial communities, especially those that produce SCFAs, are preferentially stimulated by prebiotics, which are indigestible feed ingredients. Butyrate synthesis is increased by fibres and oligosaccharides (such as inulin, fructooligosaccharides, and mannan-oligosaccharides), which also boost intestinal integrity and anti-inflammatory signalling (Obayomi et al., 2024). It has been demonstrated that prebiotic supplementation enhances feed efficiency, immunological responses, and resistance to enteric pathogen challenges (Davani-Davari et al., 2019).

Typical dietary inclusion levels range from 0.5% to 1.0% of feed, depending on feed formulation and bird age (Ravanal et al., 2025). These compounds are relatively inexpensive and compatible with standard feed manufacturing processes, offering a cost-effective and operationally feasible means of modulating the microbiota and immune function. In tropical environments, locally available fibre sources such as banana flour and cassava peel fibre can serve as cost-effective prebiotic substrates to promote butyrate-producing bacteria.

### 6.2 Probiotics

Probiotic supplementation introduces live beneficial microorganisms to reinforce gut microbial balance and metabolite synthesis. They alter the makeup and metabolic output of the gut microbiota. Strains of Bifidobacterium, *Bacillus*, and *Lactobacillus* improve SCFA production, competitive exclusion of pathogens, and regulation of bile acid metabolism (Latif et al., 2023).

Especially in intensive production settings, probiotic supplementation enhances resilience against oxidative stress, vaccination responses, and immunological status.

Commonly used strains include *Lactobacillus acidophilus*, *Bacillus subtilis*, and *Enterococcus faecium*, administered at levels of 10^8^–10^9^ CFU/g of feed or 10^6^–10^7^ CFU/mL in drinking water (Jha et al., 2020; Yaqoob et al., 2022; Gelinas et al., 2024). These probiotics enhance SCFA production, reduce pathogen load, and modulate inflammatory responses, thereby improving nutrient utilisation and feed efficiency.

However, strain specificity, stability during pelleting, and cost must be considered for practical implementation, particularly under tropical production conditions where temperature and humidity can affect microbial viability.

### 6.3 Synbiotics and postbiotics

By improving the colonisation of beneficial microorganisms and maximising their metabolic activity, synbiotics - combinations of probiotics and prebiotics - offer synergistic advantages. Postbiotics, which are characterised as microbial metabolites or inactivated microbial products, provide the host with immediate access to bioactive substances such as peptides, bacteriocins, or SCFAs (Rafique et al., 2023). Without relying on living microbial populations, these methods offer a controlled approach to achieving immunometabolic benefits.

### 6.4 Herbal extracts and phytochemicals

Polyphenols, essential oils, and plant extracts are examples of phytochemicals that affect the metabolic outputs and microbial composition (Akosile et al., 2023; Oni et al., 2024b; Adjei-Mensah et al., 2025). Polyphenols also act as substrates for microbial metabolism, producing bioactive compounds that boost antioxidant defences and alter immunological pathways. Compounds including thymol, carvacrol, curcumin, and tannins support bacteria that produce SCFAs while inhibiting infections (Lillehoj et al., 2018).

### 6.5 Precision nutrition strategies

Precision nutrition aims to match nutrient supply with the bird’s physiological and immunometabolic requirements at each stage of growth. This approach integrates detailed knowledge of nutrient–microbiota–immune interactions to optimise performance and welfare outcomes. Supplementation with functional amino acids (e.g., threonine, methionine) and vitamins (e.g., E, C, B-complex) enhances microbial metabolite synthesis, antioxidant defence, and immune competence. Recommended inclusion levels typically range from 5% to 10% above standard nutritional guidelines, depending on environmental stress intensity, health status, and breed characteristics (Alagawany et al., 2020; Lee and Rochell, 2022; Zhang et al., 2023c; Allam et al., 2025).

Although precision nutrition may increase formulation and monitoring costs, it yields improved feed efficiency, immune resilience, and welfare outcomes, particularly when implemented through sensor-based or automated feeding systems that allow real-time adjustment of nutrient supply. Both the host and its gut microbiota benefit from optimised nutrient availability under such customised regimens.

#### 6.5.1 Key precision nutrition interventions include

- Dietary fibre optimisation: Modifying the type and particle size of dietary fibre enhances microbial fermentation efficiency, promoting beneficial SCFA production and mucosal health (Yao et al., 2022).
- Amino acid balance: Ensuring adequate levels of arginine and glutamine supports mucosal immunity, microbial symbiosis, and immune cell metabolism (Cruzat et al., 2018).
- Fat modulation: Regulating dietary fat quantity and quality influences microbial modification of bile acid pools, with downstream effects on metabolic and immune signalling (Hamamah et al., 2023).

These strategies contribute to integrated immunometabolic regulation, enabling poultry to maintain homeostasis and productivity under diverse rearing and environmental conditions.

#### 6.5.2 Feasibility and regional applicability

The adoption of these strategies depends on cost-effectiveness, feed resource availability, and climatic conditions. In tropical or resource-limited regions, locally available prebiotic sources (e.g., banana flour, cassava peel fibre) and thermostable probiotic formulations can serve as viable, low-cost alternatives to commercial products. Integrating these approaches within climate-smart feeding programs enhances both sustainability and animal welfare.

Precision nutrition aligns nutrient supply with immunometabolic demand, supporting microbial metabolite synthesis and host resilience. Real-time nutrient adjustments using automated or sensor-based feeding systems can further refine this approach in commercial settings.

### 6.6 Management approaches

In addition to dietary manipulations, management techniques influence microbiota and their byproducts.

- Early-life microbial programming: Metabolite synthesis trajectories are shaped by early exposure to advantageous bacteria, such as through hatchery probiotics in ovo supplementation (Shehata et al., 2021; Gao et al., 2024).
- Stress reduction: Microbial diversity and metabolite synthesis are preserved by reducing stocking density, heat stress, and handling stress (Insawake et al., 2025).
- Litter management: By keeping litter conditions clean, beneficial microbial activity is supported and dysbiosis is decreased (Dančová et al., 2025).

## 7 Applications in poultry health and productivity

Utilising metabolites produced by the gut microbiota presents a viable strategy to enhance poultry resilience, health, and productivity. By acting as mediators to match immunological competence with metabolic and growth needs, these metabolites lessen the need for antibiotics while promoting sustainability.

### 7.1 Enhancing disease resistance

The host’s defences against systemic and intestinal infections are reinforced by microbial metabolites.

- SCFAs decrease susceptibility to *Escherichia coli* and *Salmonella* by lowering gut pH, preventing pathogen colonisation, and enhancing barrier integrity (Liu et al., 2021).
- Tryptophan metabolism produces indole derivatives, which strengthen mucosal defences and increase tolerance to commensal microorganisms (Pei et al., 2025).
- Polyamines and peptides that resemble bacteriocins have direct antibacterial action.

Together, these processes lower the pathogen burden and improve the health of the poultry flock (Lagha et al., 2017). These effects translate to measurable improvements in performance traits, including reduced mortality, improved average daily gain, and lower feed conversion ratios.

### 7.2 Supporting vaccine responses

The effectiveness of vaccines is enhanced by the regulation of immunometabolism through microbial metabolites.

- Following vaccination, SCFAs increase antibody titers by promoting regulatory and effector T cell responses (Park et al., 2014).
- Gut microorganisms create vitamins like riboflavin and folate, which promote lymphocyte proliferation and are essential for vaccine-induced immunity (Liu et al., 2024).

This implies that approaches targeting the microbiome can be used in conjunction with commercial poultry immunisation programs.

### 7.3 Mitigating heat stress and oxidative stress

Heat stress adversely affects the immune system, damages the intestines, and takes energy away from development.

- Under heat stress, butyrate reduces leaky gut by supporting tight junction proteins (Ncho, 2025). It has been shown that *Faecalibacterium* spp. is a crucial microbe in maintaining colonic mucosal health due to its high butyrate production and its roles in regulating tight junction gene expression (Heinken et al., 2014).
- Acetate and propionate offer substitute energy substrates when metabolic changes caused by stress occur (Qaid and Al-Garadi, 2021).
- Microbial antioxidants, such as metabolites of vitamin B, enhance redox equilibrium and shield immune cells from apoptosis brought on by stress (Oke et al., 2024).

Practical application of microbial metabolites or their precursors has demonstrated measurable reductions in corticosterone levels and intestinal oxidative damage in heat-stressed broilers.

### 7.4 Improving gut health and feed efficiency

Metabolites function at the nexus of immunity, digestion, and nutrition. Feed efficiency in poultry and other livestock is strongly influenced by the integrity and inflammatory status of the gut (Ducatelle et al., 2023). Chronic, low-grade intestinal inflammation, often triggered by dietary antigens, behavioural or environmental stressors, and microbial imbalance, can divert nutrients away from growth toward immune responses, thereby compromising nutrient utilisation efficiency. Historically, the observed improvement in feed efficiency with subtherapeutic antibiotic use (e.g., macrolides and tetracyclines) was attributed not only to antimicrobial activity but also to their anti-inflammatory properties, which alleviated gut inflammation and improved nutrient absorption efficiency (Alexander et al., 2008; Plata et al., 2022; Wiesner et al., 2024).

Emerging evidence indicates that microbiota-derived metabolites can provide similar physiological benefits through non-antibiotic mechanisms.

Short-chain fatty acids (SCFAs) enhance nutrient absorption by providing energy substrates for enterocytes and maintaining gut barrier integrity (Alexander et al., 2019).

Amino acid–derived metabolites regulate nitrogen metabolism and optimise protein utilisation (Qaid and Al-Garadi, 2021). By modulating gut inflammation, reinforcing mucosal health, and promoting microbial balance, these metabolites collectively enhance feed efficiency while reducing reliance on antibiotic growth promoters. This integrated approach supports both productivity and sustainability in modern livestock systems.

### 7.5 Reducing antibiotic reliance and promoting sustainability

The global shift toward antibiotic-free poultry production systems underscores the importance of harnessing microbiota-derived metabolites as natural immunomodulators and performance enhancers. Traditionally, antibiotic growth promoters (AGPs) improved productivity primarily by reducing subclinical inflammation and modulating intestinal microbial balance (Omonijo et al., 2025). However, growing concerns about antimicrobial resistance (AMR), regulatory restrictions, and consumer preferences for “antibiotic-free” products have necessitated the search for safer, sustainable alternatives.

Microbiota-derived metabolites, including short-chain fatty acids (SCFAs), bile acid derivatives, polyamines, and indole compounds, exert broad-spectrum physiological effects that mimic several beneficial actions of AGPs. By activating receptors such as FFAR2/3, HCAR2, FXR, and TGR5, these metabolites regulate inflammatory tone, strengthen intestinal barrier integrity, and maintain microbial homeostasis. For instance, butyrate and propionate not only enhance epithelial tight-junction protein expression but also suppress pro-inflammatory cytokines (e.g., TNF-α, IL-6) and stimulate the production of anti-inflammatory mediators such as IL-10 (Parada Venegas et al., 2019). This immune modulation enhances nutrient absorption and growth performance in broiler chickens. In addition to these endogenous metabolites, dietary interventions using organic acids, prebiotics, probiotics, and postbiotics can further augment host–microbiota interactions. For example, boric acid supplementation significantly reduced *Salmonella* enteritidis colonisation and alleviated the detrimental effects of necrotic enteritis in broilers (Hernandez-Patlan et al., 2019). Such outcomes demonstrate the potential of targeted metabolite modulation in disease control without reliance on antibiotics. Likewise, supplementation with butyrate-producing bacterial strains or precursors enhances gut barrier repair following coccidial challenge and promotes early immune maturation.

By modulating gut inflammation, reinforcing mucosal health, and promoting microbial balance, microbiota-derived metabolites collectively improve feed efficiency and reduce pathogen load. Beyond their role in growth promotion, these mechanisms align with global sustainability goals by reducing antibiotic use, lowering the risk of antimicrobial resistance, and minimising environmental contamination by decreasing drug residues in poultry manure. Moreover, metabolite-driven nutritional strategies can be integrated into climate-smart production frameworks, enhancing bird resilience to heat stress and disease while ensuring food safety and consumer trust.

## 8 Emerging tools and technologies

Recent advances in analytical platforms, computational biology, and experimental models are transforming how poultry scientists study and exploit gut microbiota-derived metabolites. These innovations move the field beyond descriptive studies toward predictive, mechanistic, and precision-oriented applications. Together, they are enabling a systems-level understanding of host–microbiota–metabolite interactions, paving the way for more sustainable, health-oriented poultry production.

### 8.1 Omics approaches

The advent of multi-omics technologies has revolutionised the ability to characterise microbial communities and their metabolic outputs in poultry.

- Metagenomics employs next-generation sequencing to uncover the taxonomic composition and functional genes of the gut microbiota. Beyond identifying microbial taxa, metagenomic annotation of metabolic pathways (e.g., SCFA synthesis, bile acid conversion, and amino acid fermentation) allows researchers to predict microbial contributions to host physiology and feed efficiency.
- Metatranscriptomics captures the *active* transcriptional landscape of gut microbes, providing insight into which genes are being expressed in real time under specific dietary or environmental conditions.
- Metabolomics, through platforms such as nuclear magnetic resonance (NMR) and mass spectrometry (MS), profiles hundreds of small molecules, including SCFAs, bile acids, indole derivatives, and amino acid metabolites, in intestinal digesta, serum, or excreta. These data link microbial activity to host metabolic status and health outcomes.
- Proteomics and transcriptomics of host tissues reveal how microbial metabolites modulate protein expression, enzyme activity, and signalling pathways in the gut, liver, or immune organs.

When multi-omics integration is applied (e.g., combining metagenomic, metabolomic, and transcriptomic datasets), researchers can map causal host–microbe–metabolite networks. Such integration helps identify molecular biomarkers and therapeutic targets for enhancing nutrient utilisation, stress resilience, and disease resistance in poultry.

For poultry metabolomics, the most informative sample matrices are caecal content, ileal digesta, serum, and liver tissue. Targeted and untargeted LC–MS or GC–MS methods using internal standards are recommended. Integration with 16S or shotgun metagenomics enables simultaneous mapping of microbial composition and metabolite function.

### 8.2 Gnotobiotic and germ-free models

Germ-free poultry models, raised in sterile environments, provide a controlled framework for examining the direct effects of microbiota and their metabolites on host development, immunity, and metabolism. These models isolate microbial effects from other confounding variables, allowing for clear causal inference.

Gnotobiotic models, inoculated with defined microbial consortia, go a step further by enabling the manipulation of specific bacterial species or metabolic functions. For instance, colonising birds with butyrate-producing strains allows assessment of how SCFA production modulates gut integrity or immune cell differentiation. Despite the technical demands, these systems represent powerful platforms for validating findings from omics studies and for developing next-generation probiotics or postbiotics with targeted physiological benefits.

### 8.3 Biomarker discovery and precision applications

The identification of microbial or metabolite biomarkers offers new frontiers in precision poultry management. Circulating or faecal metabolites such as bile acids, indoles, tryptophan metabolites, and SCFAs (notably butyrate and propionate) are being explored as non-invasive indicators of gut health, feed conversion efficiency, and immune competence.

Advanced metabolomics and bioinformatics now enable early biomarker detection to predict stress responses, disease susceptibility, or suboptimal nutrient absorption. This capability aligns with precision poultry farming, where sensor-driven data on metabolite profiles could inform real-time dietary adjustments, health interventions, and flock management decisions—minimising reliance on antibiotics while optimising welfare and productivity. Emerging metabolite biomarkers, such as butyrate, propionate, indole-3-acetic acid, and bile acid derivatives, provide non-invasive indicators of gut health and feed efficiency. Regular profiling may guide real-time management decisions.

### 8.4 Machine learning and systems biology

Emerging computational and modelling tools are enabling a data-driven revolution in poultry metabolite research. Machine learning algorithms and systems biology frameworks integrate complex omics, physiological, and environmental datasets to predict functional outcomes.

- Machine learning can identify predictive metabolite signatures associated with heat stress tolerance, disease vulnerability, or superior feed efficiency.
- Network modelling and dynamic simulations reveal feedback loops among diet composition, microbial metabolism, and host responses, guiding hypothesis-driven experimentation.
- Ultimately, these tools support precision nutrition design, where rations are optimised to favour beneficial microbial pathways and metabolite production tailored to genetic lines, production systems, or climatic conditions.

These approaches could move the field toward next-generation, intelligent poultry production systems that leverage microbiome and metabolite data to achieve sustainability, resilience, and welfare enhancement.

## 9 Future perspectives and research gaps

Foundational studies on the energetics of immune function on the microbiome–immune–metabolic interface have already established poultry-specific models for immunometabolic integration. These pioneering works provided the conceptual framework for understanding how nutrient status, microbial activity, and immune responses are metabolically interconnected in birds. Building on this legacy, future research should expand these frameworks through multi-omics integration, causal experimentation, and system-level modelling to clarify the molecular mechanisms of metabolite–immune interactions.

Despite growing evidence that metabolites originating from the gut microbiota are essential for controlling poultry immunometabolism, the field still lacks standardised protocols, quantitative mapping, and cross-validation of findings across production systems. Addressing these knowledge gaps will be crucial for developing practical, metabolite-driven interventions that enhance poultry health, stress resilience, and sustainable production.

Despite growing evidence that metabolites originating from the gut microbiota are essential for controlling poultry immunometabolism, the field still lacks standardised protocols, quantitative mapping, and cross-validation of findings across production systems. Addressing these knowledge gaps will be crucial for developing practical, metabolite-driven interventions that enhance poultry health, stress resilience, and sustainable production. The knowledge gaps and future research priorities in poultry immunometabolism are summarised in Table 3.

**TABLE 3:** Knowledge gaps and future research priorities in poultry immunometabolism.

Area	Current gap	Research priority	References
Metabolite–immune mapping	Most data extrapolated from mammals; poultry-specific evidence is limited	Systematic mapping of metabolite–immune pathways in poultry	Oladosu et al. (2023)
Metabolite quantification	Lack of standardised protocols across studies	Develop validated, reproducible metabolomics pipelines for poultry	Zhang, et al. (2024)
Emerging metabolites	Focus mainly on SCFAs and bile acids	Explore polyamines, microbial peptides, and novel indole derivatives	Bi et al. (2022)
Causal studies	Predominantly correlational findings	Employ germ-free/gnotobiotic models and targeted metabolite supplementation	Tian et al. (2023)
Integration into farming systems	Limited application in precision poultry farming	Combine microbiome modulation with digital tools (sensor-based welfare monitoring)	An et al. (2025)
Vaccine response links	Poorly understood connection between metabolites and vaccine efficacy	Investigate immunometabolic priming to enhance vaccine outcomes	Vuscan et al. (2024)

### 9.1 Limited poultry-specific data

Mammalian research provides a large portion of the present knowledge regarding immunometabolism and microbial metabolites. The distinct digestive physiology of poultry species, such as their crop, gizzard, and shorter intestinal transit time, may change the metabolic dynamics of microorganisms. Poultry-specific mechanistic research is needed to confirm results from other animal models.

### 9.2 Metabolite measurement standardisation

Comparability between research is hindered by the absence of standardised procedures for data reporting, metabolite quantification, and sampling. The field will be strengthened by the development of approved reference techniques for quantifying bile acids, SCFAs, and metabolites produced from amino acids in poultry.

### 9.3 Unexplored and novel metabolites

While additional metabolites (such as polyamines, microbial peptides, and conjugated fatty acids) are still poorly understood, the majority of studies have been on SCFAs, bile acids, and amino acid derivatives. One study frontier is the identification of novel bioactive metabolites and their association with immunological and metabolic processes in poultry.

### 9.4 Integration into precision poultry production

Future research should focus on incorporating microbiome manipulation into precision farming frameworks, employing biomarkers, omics, and machine learning to predict and optimise metabolite-driven outcomes. Microbial metabolites are rarely considered as functional outputs in current poultry nutrition and management programs.

### 9.5 Antibiotic alternatives and sustainable production

Microbiota-derived metabolites offer a promising substitute to preserve flock health and productivity as worldwide antibiotic prohibitions persist; however, extensive field validation and cost-benefit evaluations are required before the widespread use of metabolite-focused interventions.

### 9.6 Risks, trade-offs, and safety considerations

Not all microbial metabolites are beneficial at all concentrations. Excessive butyrate may reduce feed intake, while certain secondary bile acids and indole derivatives can disrupt barrier integrity if accumulated excessively. Interactions with vaccines, ionophores, or antibiotics should be monitored to prevent interference with immune priming. Regulatory assessment of metabolite-based additives remains essential to ensure compliance with residue and food-safety requirements.

## 10 Conclusion

Metabolites generated by the gut microbiota have emerged as key modulators of poultry immunometabolism, integrating host physiology, microbial activity, and nutrition. Compounds such as microbial vitamins, bile acid derivatives, short-chain fatty acids, and amino acid metabolites regulate essential functions, including immune regulation, energy balance, oxidative stress resilience, and intestinal barrier maintenance. Acting as both nutrients and signalling molecules, these metabolites bridge the immune and metabolic systems that sustain poultry productivity and resilience.

Strategic modulation of microbial metabolites through nutritional and management interventions offers a practical route to enhancing bird health and reducing dependence on antibiotics. When complemented by improved husbandry practices and functional feed innovations, such as postbiotics, phytochemicals, precision feeding, prebiotics, synbiotics, and probiotics, these strategies can optimise microbial metabolic efficiency and host immune competence. Moreover, the integration of emerging research tools, including multi-omics platforms, biomarker discovery, and machine learning, now provides unprecedented opportunities for targeted manipulation and monitoring of metabolite-driven outcomes.

Gut microbiota-derived metabolites, therefore, represent a promising frontier for sustainable and antibiotic-free poultry production. Future research should prioritise poultry-specific mechanistic studies, standardised metabolite quantification techniques, and integrative multi-omics approaches to translate these mechanistic insights into practical, field-level applications that enhance productivity, welfare, and environmental sustainability.
